# Impact of small diameter and low level of emission laser coronary atherectomy in patients with acute myocardial infarction

**DOI:** 10.1007/s10103-021-03405-y

**Published:** 2021-08-26

**Authors:** Ryo Masuda, Takashi Shibui, Yoshiaki Mizunuma, Shogo Yoshikawa, Kosuke Takeda, Hirofumi Kujiraoka, Koichiro Yamaoka, Tomoyuki Arai, Dai Inagaki, Takashi Kimura, Kiyotaka Yoshida, Masao Takahashi, Takeshi Kitamura, Rintaro Hojo, Takaaki Tsuchiyama, Seiji Fukamizu, Tetsuo Sasano

**Affiliations:** 1 Department of Cardiology, Tokyo Metropolitan Hiroo Hospital, 2-34-10, Ebisu, Shibuya, Tokyo, 150-0013 Japan; 2 Department of Cardiology, Toshima Hospital in the Tokyo Metropolitan Health and Hospitals Corporation, 33-1, Sakaecho, Itabashi, Tokyo, 173-0015 Japan; 3 Department of Cardiology, Tokyo Medical and Dental University Hospital, 5-45, Yushima 1-Chome, Bunkyo, Tokyo, 113-8519 Japan

**Keywords:** Laser coronary atherectomy, Acute myocardial infarction, In-hospital outcomes, Small diameter, Low level emission

## Abstract

Excimer laser coronary atherectomy (ELCA) is an effective treatment to remove intracoronary thrombi. In the present study, we compared in-hospital mortality in patients with acute myocardial infarction (AMI) who underwent conventional treatment and conventional treatment plus ELCA. Among 656 patients who were admitted to our hospital through the Tokyo CCU Network, 104 patients with AMI who were treated by percutaneous coronary intervention between January 2013 and December 2016 met inclusions criteria and underwent conventional treatment with ELCA (ELCA group) and 89 underwent conventional treatment alone (conventional group). We retrospectively evaluated in-hospital mortality within 30 days and used propensity score (PS) matching to reduce assignment bias and multivariate analysis to detect the predictors of in-hospital mortality. In-hospital mortality rate was significantly lower in the ELCA group before and after PS matching (2.9% vs. 13.5%, *p* = 0.006 before PS matching, and 2.8% vs. 14.1%, *p* = 0.016 after PS matching). After PS matching, β-blocker or statins use, incidence of shock, Killip classification, and door-to-balloon time were not significantly different. A multivariate logistic regression analysis identified ELCA, dyslipidemia, shock, and left ventricular ejection fraction as independent predictors of in-hospital mortality (odds ratio (OR), 0.147, 95% confidence interval [CI], 0.022–0.959, *p* = 0.045; OR, 0.077, 95% CI, 0.007–0.805, *p* = 0.032; OR, 6.494, 95% CI, 1.228–34.34, *p* = 0.028; OR, 0.890, 95% CI, 0.828–0.957, *p* = 0.002, respectively). Our data indicate that ELCA with the small diameter and low level emission may reduce the in-hospital mortality compared to conventional methods in patients with AMI in drug-eluting stent era.

## Introduction

Acute myocardial infarction (AMI) is usually treated with drug-eluting stent (DES) implantation [1], although sometimes intracoronary thrombus is removed through manual aspiration to prevent distal emboli before stenting [2]. In the previous study, the utilization of manual aspiration catheters has demonstrated a positive impact on myocardial perfusion and late clinical outcome [3]. However, manual thrombus aspiration has limited efficacy in AMI with thrombus rich lesion. The main cause of procedural failure is insufficient thrombus removal. Another study was showing that it increases the risk of stroke [4]. Consequently, another capable technology for improved thrombus removal is highly needed [5]. ELCA is one of the most effective treatments to remove intracoronary thrombi [6]. Laser atherectomy in AMI presents several advantages: rapid elimination of the thrombus with vaporization, removal of procoagulant reactants, diminution of the risk of distal embolization, and reduction of platelet aggregation promotors (“stunned platelets” phenomenon) [7, 8]. Hence, the safety and capable thrombus removal technology of ELCA in patients with AMI was revived in recent few years especially in Japan [9]. In addition, ELCA has also been applied to a variety of difficult-to-treat lesions, including preparation before drug-coated balloon angioplasty [10]. ELCA improves thrombolysis in myocardial infarction (TIMI) frame count or myocardial blush grade (MBG) in patients with AMI [11–13]. However, clinical data supporting the utilization of ELCA in AMI remain scarce [14, 15]. This study aimed to compare in-hospital outcome in patients with AMI between conventional treatment and conventional treatment plus ELCA in the contemporary era of percutaneous coronary intervention (PCI).

## Methods

### Study population

**Fig. 1:**
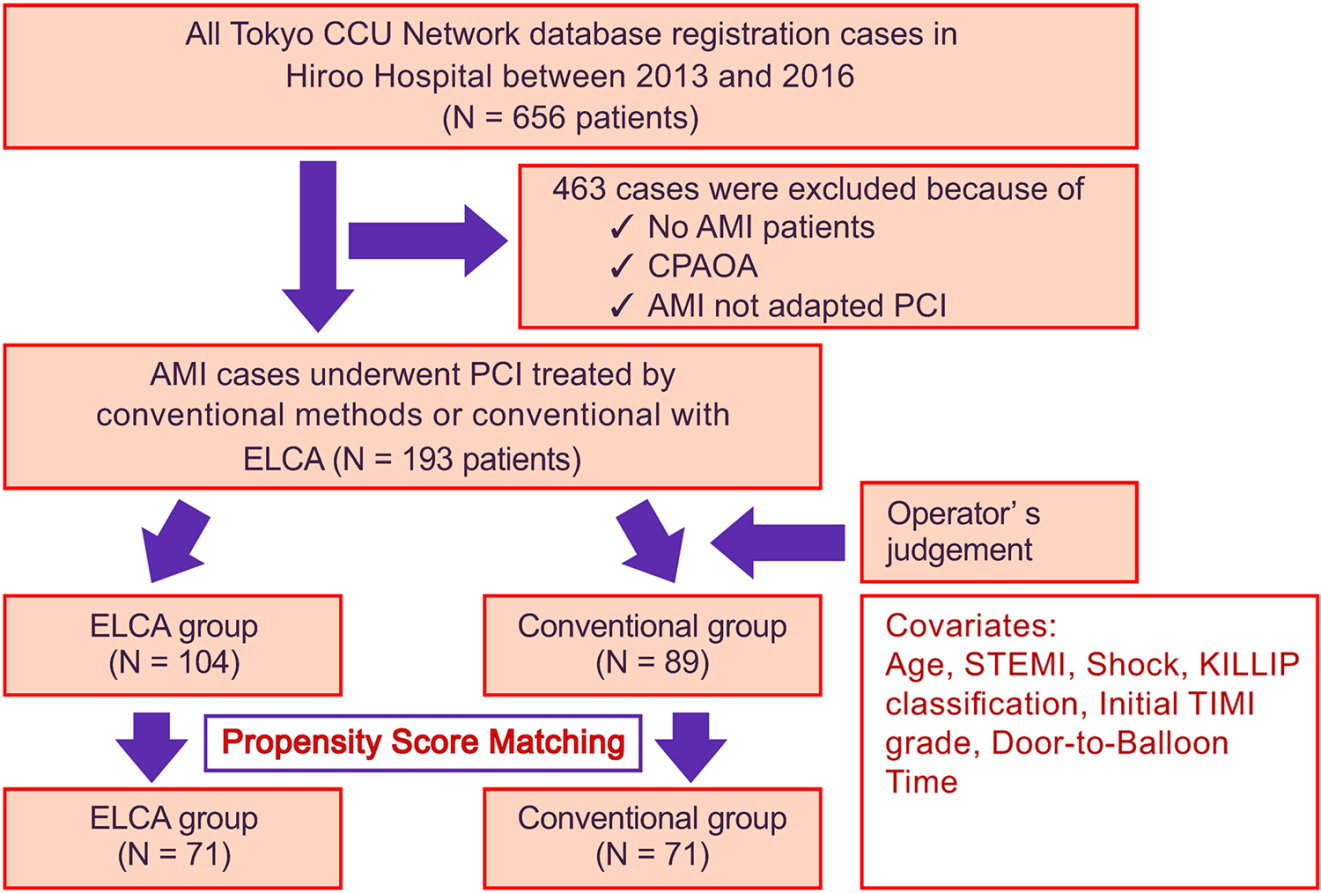
Flow diagram of this study. The ELCA group received conventional treatment with ELCA, while the remaining patients were treated using conventional methods (conventional group). CPAOA, cardiopulmonary arrest on arrival; AMI, acute myocardial infarction; PCI, percutaneous coronary intervention; ELCA, excimer laser coronary atherectomy; TIMI, thrombolysis in myocardial infarction; STEMI, ST-elevation myocardial infarction

**Fig. 2:**
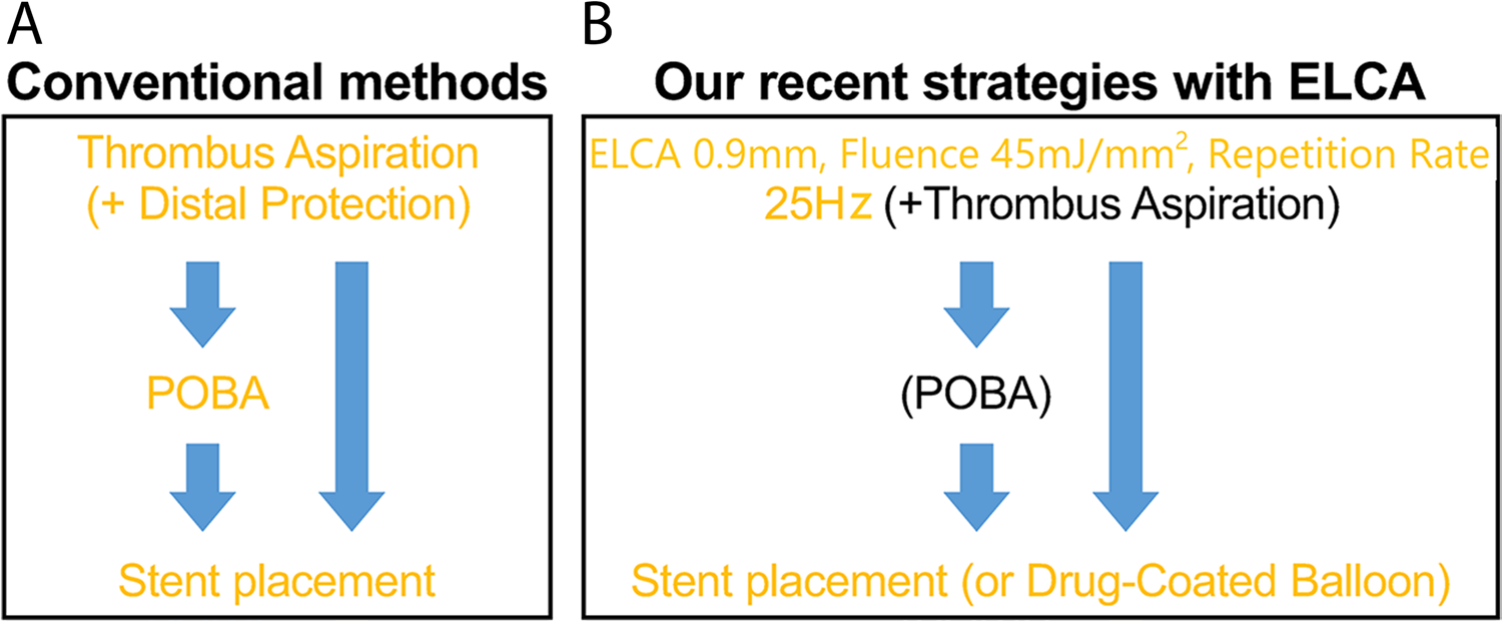
Definition of PCI strategies in acute myocardial infarction. **A** Conventional methods and **B** our recent strategies with ELCA. ELCA, excimer laser coronary atherectomy; POBA, plain old balloon angioplasty

Among 656 patients who were admitted to our hospital through the Tokyo CCU Network, 193 patients with AMI who underwent PCI between January 2013 and December 2016 were evaluated. AMI was diagnosed on the basis of universal definition of myocardial infarction [16]. AMI with cardiogenic shock was confirmed by clinical criteria. The clinical criteria were hypotension on admission (a systolic blood pressure of < 90 mm Hg or the need for supportive catecholamines or mechanical supports to maintain a systolic blood pressure of ≥ 90 mm Hg). Patients with cardiopulmonary arrest on arrival were excluded. In this study, AMI included both ST segment elevation myocardial infarction (STEMI) and non-ST segment elevation myocardial infarction (NSTEMI). The reasons are as follows. First, in a study to examine the relationship between the thrombectomy effect of ELCA and outcome, it was considered that there was no need to separate them because they were the same occurring mechanism due to thrombus associated with plaque rupture or erosion. However, unstable angina without elevation of cardiac troponin was excluded because it may have a favorable prognosis. Second, STEMI is difficult to analyze because of the small number of cases. Third, the earlier CARMEL multicenter registry did not separate the two [14]. A total of 104 patients underwent conventional treatment with ELCA (ELCA group) and 89 underwent conventional methods (conventional group) (Fig. 1). The timing of PCI was decided according to the AHA/ACC guidelines [17, 18]. Six patients with NSTEMI (4 patients in the conventional group, 2 patients in the ELCA group) were undergoing PCI within 25 to 72 h of admission, and have pending improvement of pulmonary congestion due to heart failure. And all the other patients were undergoing PCI within 24 h. Propensity score (PS) matching was used to reduce treatment assignment bias. After PS matching, 71 patients in the conventional group and 71 in the ELCA group were evaluated. Conventional methods were defined as standard PCIs, including thrombus aspiration and distal protection (Fig. 2A). All ELCA procedures were performed using the Spectranetics apparatus (CVX-300 platform, Spectranetics, Colorado Springs, CO, USA). It is composed of a generator of excimer laser (CVX-300) and pulsed xenon-chloride laser catheters able to deliver laser emission from 45 to 60 mJ/mm^2^ (fluence) at pulse repetition rates of 25 to 40 Hz. The operators determined the excimer laser catheters which size was suitable concentric 0.9 or 1.4 mm judging from the result of the angiographic and intracoronary imaging. Based on our previous study, we have a tendency to use the smaller 0.9-mm excimer laser catheter (Fig. 2B) [13].

### Outcomes

We retrospectively compared in-hospital mortality within 30 days between the two groups. Furthermore, we determined the predictors of in-hospital mortality within 30 days using PS matching.

### Statistical analysis

Categorical data are expressed as percent with the number of samples in parentheses. Continuous data are expressed as mean ± standard deviation (SD). Categorical data were analyzed using the chi-squared test or Fisher’s exact test. Between-group comparison of the continuous data was performed using the unpaired *t* test. All tests were two-sided and considered significant when *p* value was inferior to 0.05. Covariates of PS calculation used age, ST-elevation myocardial infarction, shock, Killip classification [19], initial TIMI grade [20], and door-to-balloon time. We used multivariate logistic regression analysis to estimate the predictors of in-hospital mortality. All statistical analyses were performed using the 17th version of the software SPSS (IBM SPSS Statistics, IBM, Armonk, NY, USA).

#### Ethical approval/informed consent

This study was conducted in accordance with the principles of the Declaration of Helsinki and in compliance with the International Conference on Harmonization—Good Clinical Practice and local regulatory requirements. The study was approved by the Tokyo Metropolitan Hiroo Hospital ethics committee. All patients provided written informed consent to receive ELCA treatment and have their data used this study.

## Results

**Fig. 3:**
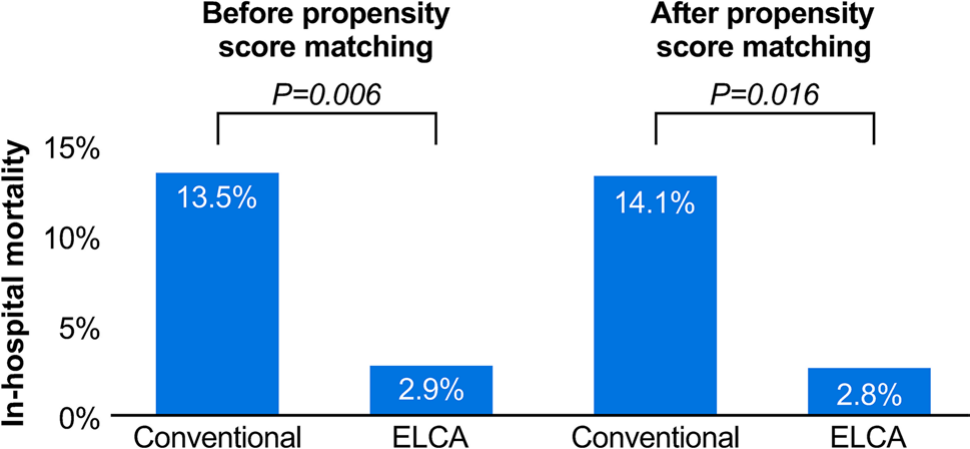
In-hospital mortality within 30 days in patients suffering AMI**.** In-hospital mortality rate in percent within 30 days in patients suffering acute myocardial infarction (AMI) treated by conventional methods or conventional methods and excimer laser coronary atherectomy (ELCA) before and after propensity score matching

**Fig. 4:**
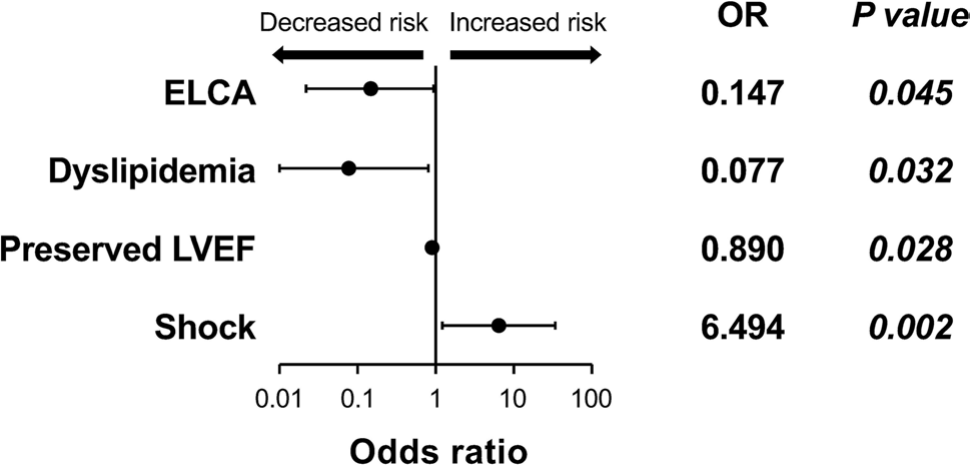
Forest plot representing the odds ratios for risk of in-hospital mortality. Forest plot showing odds ratios (ORs) for significant predictors of in-hospital mortality that were identified by multivariate logistic regression analysis. ELCA, excimer laser coronary atherectomy; LVEF, left ventricular ejection fraction

Table 1 shows baseline clinical characteristics of the two groups. After PS matching, we detected no significant differences between the two groups concerning β blockers or statins use, incidence of shock, Killip classification, symptom onset-to-door time, and door-to-balloon time (Table 1). The mean laser catheter diameter, maximum fluence, and repetition rate were 1.08 mm, 51.1 mJ/mm^2^, and 31.3 Hz, respectively. In 10.9% of the cases, the fluence and repetition rate were increased due to poor improvement in coronary blood flow. In-hospital mortality rate (all mortality cases were cardiovascular deaths that were not caused by PCI procedures or ELCA-related devices) was significantly lower in the ELCA group than the conventional group before and after PS matching (2.9% vs. 13.5%, *p* = 0.006 before PS matching, and 2.8% vs. 14.1%, *p* = 0.016 after PS matching) (Fig. 3). A multivariate logistic regression analysis identified ELCA (odds ratio (OR), 0.147; 95% confidence interval [CI], 0.022–0.959; *p* = 0.045), dyslipidemia (OR, 0.077; 95% CI, 0.007–0.805; *p* = 0.032), left ventricular ejection fraction (OR, 0.890; 95% CI, 0.828–0.957; *p* = 0.002), and shock (OR, 6.494; 95% CI, 1.228–34.34; *p* = 0.028) as independent predictors of in-hospital mortality (Tables 2 and 3 and Fig. 4). Moreover, ELCA reduced in-hospital mortality rate by 87% as analyzed using inverse PS weighting (OR, 0.227; 95% CI, 0.060–0.863; *p* = 0.030).

**Table 1:** Baseline clinical characteristic of patients

	**Before propensity score matching**			**After propensity score matching**		
	Conventional	ELCA	*p* value	Conventional	ELCA	*p* value
Variables	(*n* = 89)	(*n* = 104)		(*n* = 71)	(*n* = 71)	
Age years	69.91 ± 14.48	67.35 ± 13.77	0.209	68.52 ± 14.67	67.9 ± 14.17	0.798
Sex category, male, %	63 (70.8)	80 (76.9)	0.322	51 (71.8)	55 (77.5)	0.440
Smoker, %	43 (48.9)	56 (53.8)	0.491	36 (50.7)	39 (54.9)	0.614
Diabetes, %	28 (31.5)	27 (26.0)	0.399	24 (33.8)	20 (28.2)	0.468
Hypertension, %	56 (62.9)	55 (52.9)	0.160	46 (64.8)	39 (54.9)	0.231
Dyslipidemia, %	41 (46.1)	51 (49.0)	0.680	34 (47.9)	36 (50.7)	0.737
Antiplatelets use, %	24 (27.0)	24 (23.1)	0.533	20 (28.2)	15 (21.1)	0.330
Statins use, %	23 (25.8)	22 (21.2)	0.443	18 (25.4)	13 (18.3)	0.310
ACEs / ARBs use, %	29 (32.6)	25 (24.0)	0.187	23 (32.4)	18 (25.4)	0.355
β blockers use, %	13 (14.6)	15 (14.4)	0.971	12 (16.9)	9 (12.7)	0.478
STEMI, %	63 (70.8)	79 (76.0)	0.416	54 (76.1)	54 (76.1)	1.000
LVEF, %	49.7 ± 13.23	50.57 ± 12.57	0.632	48.8 ± 13.3	49.1 ± 12.7	0.904
Shock, %	12 (13.5)	15 (14.4)	0.851	11 (15.5)	10 (14.1)	0.813
Door-to-balloon time, min	676.51 ± 173.21	146.63 ± 31.11	*0.003*	147.92 ± 15.5	136.4 ± 19.1	0.640
Onset-to-door time, min	448.86 ± 571.81	506.63 ± 1119.03	0.705	435.58 ± 581.80	523.75 ± 1329.09	0.652
MI location						
Inferior, %	36 (40.4)	37 (35.6)	0.318	28 (39.4)	24 (33.8)	0.208
Posterior, %	6 (6.7)	8 (7.7)		5 (7.0)	5 (7.0)	
Anterior, %	43 (48.3)	47 (45.2)		34 (47.9)	34 (47.9)	
Lateral, %	4 (4.5)	12 (11.5)		4 (5.6)	8 (11.3)	
Troponin I μg/ml	32.83 ± 10.6	53.12 ± 17.7	0.346	37.58 ± 13.1	43.9 ± 20.2	0.792
KILLIP classification						
I or II, %	72 (80.9)	88 (84.6)	0.494	56 (78.9)	56 (78.9)	1.000
III or IV, %	17 (19.1)	16 (15.4)		15 (21.1)	15 (21.1)	
Initial TIMI grade						
0, %	44 (50.0)	45 (43.3)	0.641	37 (52.1)	40 (56.3)	0.740
1, %	7 (8.0)	6 (5.8)		7 (9.9)	4 (5.6)	
2, %	15 (17.0)	20 (19.2)		11 (15.5)	9 (12.7)	
3, %	22 (25.0)	33 (31.7)		16 (22.5)	18 (25.4)	
Final TIMI grade						
0, %	2 (2.2)	0 (0.0)	0.311	2 (2.8)	0 (0.0)	0.328
1, %	1 (1.1)	1 (1.0)		1 (1.4)	1 (1.4)	
2, %	1 (1.1)	0 (0.0)		1 (1.4)	0 (0.0)	
3, %	85 (95.5)	103 (99.0)		67 (94.4)	70 (96.5)	

Data are presented as *n* (%), except for age, left ventricular ejection fraction (LVEF), door-to-balloon time, onset-to-door time, and troponin I level, which are presented as mean ± standard deviation. *ARBs* angiotensin II receptor blockers; *ACEs* angiotensin converting enzyme inhibitors; *STEMI* ST-elevation myocardial infarction; *MI* myocardial infarction; *TIMI* thrombolysis in myocardial infarction; *ELCA* excimer laser coronary atherectomy

**Table 2:** Predictors of in-hospital mortality identified by univariate and multivariate logistic regression analyses, before propensity score matching

	Univariate analysis			Multivariate analysis		
	OR	95% CI	*p* value	OR	95% CI	*p* value
Age	1.052	1.006–1.101	0.026	1.068	1.009–1.131	0.024
Sex category, male	0.364	0.125–1.062	0.064			
Current smoker	0.314	0.096–1.023	0.055			
Diabetes	1.280	0.417–3.932	0.666			
Hypertension	0.722	0.232–2.250	0.574			
Dyslipidemia	0.250	0.068–0.916	0.036			
Statins use (pre-PCI)	1.215	0.367–4.020	0.750			
ACEs/ARBs use (pre-PCI)	0.931	0.283–3.060	0.906			
Anterior MI	0.746	0.255–2.184	0.593			
LVEF	0.909	0.866–0.954	< 0.001	0.915	0.866–0.968	*0.012*
Cardiogenic shock	9.564	3.119–29.324	< 0.001	12.261	1.693–88.821	*0.013*
Door-to-balloon time	1.000	1.000–1.000	0.599			
Cardiac troponin I	1.002	1.000–1.004	0.119			
KILLIP classification (> = III)	9.625	3.144–29.469	< 0.001	7.635	1.376–42.373	*0.020*
Initial TIMI grade	0.674	0.424–1.071	0.095			
Final TIMI grade	0.539	0.227–1.278	0.161			
ELCA	0.191	0.052–0.699	0.012	0.176	0.039–0.792	*0.024*

**Table 3:** Predictors of in-hospital mortality identified by univariate and multivariate logistic regression analyses**,** after propensity score matching

	Univariate analysis			Multivariate analysis		
	OR	95% CI	*p* value	OR	95% CI	*p* value
Age	1.039	0.991–1.089	0.110			
Sex category, male	0.653	0.184–2.313	0.509			
Current smoker	0.269	0.069–1.037	0.057			
Diabetes	1.125	0.320–3.953	0.854			
Hypertension	0.446	0.134–1.483	0.188			
Dyslipidemia	0.080	0.010–0.641	0.017	0.077	0.007–0.805	*0.032*
Statins use (pre-PCI)	1.214	0.308–4.788	0.781			
ACEs/ARBs use (pre-PCI)	0.467	0.098–2.229	0.339			
Anterior MI	0.760	0.229–2.517	0.653			
LVEF	0.888	0.835–0.943	< 0.001	0.890	0.828–0.957	*0.002*
Cardiogenic shock	11.600	3.243–41.496	< 0.001	6.494	1.228–34.34	*0.028*
Door-to-balloon time	0.999	0.994–10..4	0.622			
Cardiac troponin I	1.002	1.000–1.005	0.074			
KILLIP classification (> = III)	9.818	2.717–35.483	< 0.001			
Initial TIMI grade	0.733	0.430–1.250	0.255			
Final TIMI grade	0.555	0.232–1.326	0.185			
ELCA	0.177	0.037–0.839	0.029	0.147	0.022–0.959	*0.045*

The odds ratio (OR) and 95% confidence interval (CI) are shown. Categories with a significant difference based on the univariate analysis were used as covariates for multivariate analysis. *ARBs* angiotensin II receptor blockers; *ACEs* angiotensin converting enzyme inhibitors; *MI* myocardial infarction; *LVEF* left ventricular ejection fraction; *TIMI* thrombolysis in myocardial infarction; *ELCA* excimer laser coronary atherectomy

## Discussion

In the present study, patients with AMI who underwent small diameter ELCA (i.e., 1.4 mm or less) and low level fluence (around 50 mJ/mm^2^) had a significant reduction in in-hospital mortality rate of 2.9%. The detailed mechanism is unknown because there is no significant difference in final TIMI flow. The efficiency of ELCA could be associated with its intrinsic properties. Indeed, excimer laser is a form of ultraviolet laser, which has been reported to remove or reduce atherosclerotic plaques and thrombi [21], improve microcirculation in patients with acute coronary syndrome (ACS) compared to thrombus aspiration therapy [22], and cause the “stunned platelets” phenomenon that reduce platelet aggregation [8]. Given that we previously identified a significant improvement in MBG induced by ELCA [13], an improvement in microcirculation is the most probable cause for the decrease in in-hospital mortality rate. In the previous study, it was reported that “platelet stunning” effect is dose dependent and most pronounced at high levels of laser emission such as 60 mJ/mm^2^ [6]. However, approximately 10% of the cases experienced deterioration of the TIMI flow grade when the level gradually increased from a low level to a high level of laser emission. We believed that strong acoustic shock waves, rather than vaporization of thrombus, may cause distal emboli under high level irradiation; therefore, we have recently been using a low level of fluence such as 45–55 mJ/mm^2^. Additionally, although the rate of in-hospital mortality in the conventional group in this study was 13.5%, which is higher than that of other hospitals in Japan [23], there are contributing factors, such as the fact that transport by air from remote islands accounts for approximately 40% of all ACS cases in our hospital and patients with severe shock requiring mechanical support have not been excluded in this study. Taking these into account, the 2.9% rate of in-hospital mortality in the ELCA group is staggering. Furthermore, our data may have been more effective for organized thrombi that were a little late in the onset of AMI. Finally, to change the subject, the laser catheter is expensive at \210,000 yen (approximately 2,000 USD), but the incremental cost-effectiveness ratio (ICER) calculated in this study is \454,400 (approximately 4,200 USD) per lifesaving procedure, which is an acceptable cost (Table 4).

**Table 4:** Incremental cost-effectiveness ratio of ELCA in patients with AMI in Japan

Devices		Cost
Aspiration catheter		¥40,800
Filter wire set		¥119,000
Total		¥159,800
Laser catheter		¥211,000
Incremental cost		¥51,200
	**Cost**	**Alive**
Conventional	¥159,800 × 71	61
ELCA	¥211,000 × 71	69
ICER = (¥51,200 × 71)/(69 − 91) = ¥454,400 ≈ 4,200 USD (per lifesaving)		

Cost of devices and the number of patients who are alive were presented and used to calculate the incremental cost-effectiveness ratio (ICER) per lifesaving procedure in Japanese yen (¥) and USD. Cost in USD has been converted from cost in yen using the current exchange rate and should be interpreted with caution due to possible large price disparities between devices in Japan and other countries. Abbreviations: *ELCA* excimer laser coronary atherectomy

## Conclusion

Our data indicate that ELCA with small diameter and low level emission may reduce the in-hospital mortality rate than the conventional methods in patients with AMI. ELCA is feasible and cost-effective in patients with AMI.

### Limitations

This study included only a small number of participants and retrospective observational one in only single center. STEMI and NSTEMI should have been analyzed separately, but the number of participants was small and the analysis was quite difficult. ICER should be calculated based on the cost for 1 year instead of 1 month, but this time, we assumed that there would be no difference in the cost after 1 month of survival. Precisely, the medical insurance system in each country should be considered. Multicenter randomized controlled trials are warranted to confirm this observational study in the future.

## Data Availability

Not applicable.
